# Asymptomatic Bacteriuria in Pregnancy Complicated by Pyelonephritis Requiring Nephrectomy

**DOI:** 10.1155/2018/8924823

**Published:** 2018-09-19

**Authors:** Sharon J. Kim, Pavan Parikh, Amanda N. King, Mary L. Marnach

**Affiliations:** ^1^Mayo Clinic, Department of Obstetrics and Gynecology, Rochester, MN, USA; ^2^Mayo Clinic, Department of Obstetrics and Gynecology, Division of Maternal Fetal Medicine, Rochester, MN, USA

## Abstract

Routine prenatal care in the United States includes screening for asymptomatic bacteriuria (ASB), which occurs in 2 to 7 percent of pregnant women and can cause urinary tract infection and pyelonephritis. We present the case of a pregnant woman affected by multidrug resistant Klebsiella induced ASB during her prenatal screen, which was untreated due to a repeat urine culture showing mixed flora; subsequently, the patient's postpartum course was complicated by pyelonephritis and perinephric abscess, concluding in a radical nephrectomy. Current recommendations are to treat ASB after two consecutive voided urine cultures showing the same bacterial strain in quantitative counts of =/> 10(5) colony forming units (cfu)/mL or a single-catheterized specimen with quantitative count of =/> 10(2) cfu/mL. For women with ASB in their prenatal screen or other high risk factors, consideration should be given to testing urine cultures every trimester until the completion of pregnancy to prevent the complications of persistent bacteriuria.

## 1. Introduction

Screening for asymptomatic bacteriuria (ASB) is part of routine prenatal care in the United States, as ASB occurs in 2 to 7 percent of pregnant women [1, 2]. Up to 40% of untreated pregnant women with ASB will develop a urinary tract infection (UTI), including pyelonephritis, with 80 percent risk reduction if bacteriuria is eradicated forming the basis for ACOG treatment recommendations [1–4].

In the absence of strong risk factors for recurrent or persistent bacteriuria such as sickle cell trait or renal transplantation, there is no guidance available to inform the care of other patients at moderately increased risk. These risk factors include a history of prior UTI, nulliparity, pre-existing diabetes mellitus, smoking, late presentation to care, and low socioeconomic status [3, 5–7]. Herein, we discuss the case of a woman affected by multidrug resistant Klebsiella induced ASB untreated in the antenatal period, leading to pyelonephritis and perinephric abscesses and concluding in radical nephrectomy in the postpartum period.

## 2. Case Presentation

The patient is a 30-year old now gravida 2 para 2, status post complete left nephrectomy in the setting of multidrug resistant Klebsiella urosepsis and left pyelonephritis during her immediate postpartum phase. The Anuak speaking woman immigrated from Kenya to the United States nine months prior to her second pregnancy and presented to care at 15 weeks' gestation. Her history included chronic hypertension without a previous history of UTI.

At her new obstetrical visit, a urinalysis demonstrated 4-10 white blood cells (WBC) per high power field and gram stain positivity for gram-negative bacilli and gram-positive bacilli. Urine culture yielded multidrug resistant Klebsiella pneumoniae, 10(4) to 10(5) colony forming units (cfu/mL). The organism was susceptible to quinolones, carbapenems, and piperacillin/tazobactam. An Infectious Disease consultation recommended a repeat clean catch culture with treatment using IV ertapenem if the culture showed the same organism. The repeat urine culture showed mixed flora without a specific organism identified. Because the patient remained asymptomatic, she did not have an additional gram stain or urine culture through the remainder of pregnancy.

**Figure 1:**
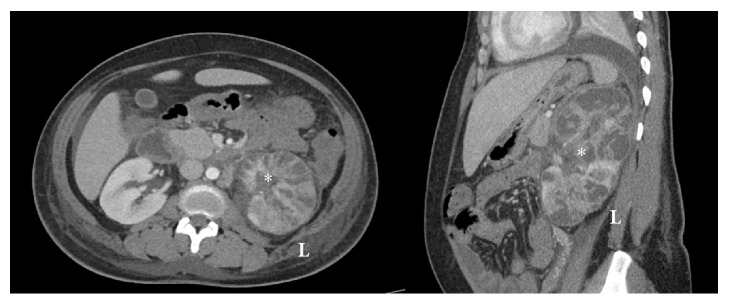
CT of abdomen, axial and sagittal views, with findings of multifocal areas of parenchymal infection and necrosis to left kidney*∗*.

**Figure 2:**
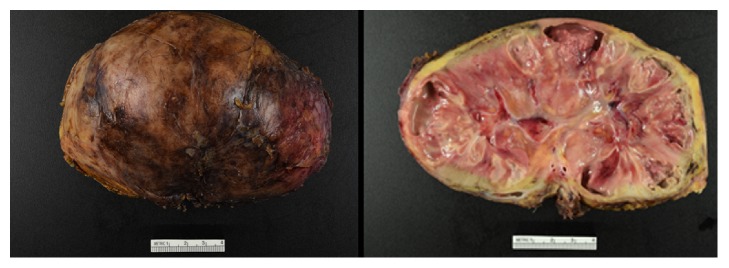
Renal parenchyma shows severe acute pyelonephritis with multifocal abscess formation and multifocal renal infarction.

At 37 weeks' gestation, the patient developed superimposed preeclampsia and underwent induction of labor with a normal spontaneous vaginal delivery without complications. On postpartum day (PPD) 0, she was afebrile but reported left sided abdominal and flank pain. A urine culture on PPD1 was positive for multidrug resistant Klebsiella/Raoultella species (sp) > 10(5) cfu/mL and sensitive to quinolones, gentamicin, and piperacillin/tazobactam. She began PO ciprofloxacin 500 mg twice daily with creatinine rising to 1.1. By PPD3, she continued to have abdominal and flank pain with creatinine rise to 1.5, and based on urine culture sensitivities, antibiotic was changed from ciprofloxacin to PO levofloxacin 500 mg daily. By PPD4 she developed new onset tachycardia, tachypnea, fever of 38.6 degrees celsius, and continued pain. As such, she was transferred to the intensive care unit with lactate 2.8 and WBC 18,000 and started on IV piperacillin/tazobactam 3.375 grams every 6 hours. A chest computerized tomography (CT) was negative for pulmonary embolism, showing a moderate left pleural effusion, and abdominal/pelvic CT compatible with pyelonephritis but no abscess. Blood cultures were positive for Klebsiella/Raoultella sp. On PPD5, when stable, she was transferred to the postpartum unit and switched to IV meropenem 500 mg every 6 hours due to persistent fevers. On PPD7, with ongoing fevers, tachycardia, and pain, a left kidney ultrasound confirmed a 5.1 cm left subcapsular abscess which was aspirated 30 cc of purulent discharge. An echocardiogram for endocarditis and HIV testing were negative. By PPD9, with continued fevers to 39.3 degrees celsius, abdominal CT imaging was concerning for left renal multifocal infection and parenchymal necrosis (Figure 1). On PPD10, after Urology and Maternal Fetal Medicine consultations, the patient underwent an open left nephrectomy. Pathology confirmed severe diffuse pyelonephritis, multifocal abscesses, and diffuse parenchymal infarction (Figure 2).

The patient recovered well thereafter with symptom resolution, creatinine 1.0, and was discharged on postoperative day 3 with a 14-day course of IV ertapenem 1 gram daily.

## 3. Discussion

While our patient's early pregnancy urine culture demonstrated multidrug resistant Klebsiella pneumonia complex at 10(4) to 10(5) cfu/ml, she was not treated antenatally due to a repeat urine culture showing mixed flora likely related to inappropriate collection technique [8]. Through the remainder of her pregnancy, she had no urinary symptoms and no additional gram stain or urine culture was obtained until complaint of left abdominal and flank pain on the night of delivery.

The immunosuppression of pregnancy, mechanical bladder compression, and ureteral dilatation facilitates the ascent of bacteria resulting in a 20-fold increased risk of pyelonephritis in gravidas [2, 7, 9, 10]. For asymptomatic women, bacteriuria is defined as two consecutive voided urine specimens with isolation of the same bacterial strain in quantitative counts > 10(5) cfu/ml or a single-catheterized urine specimen with one bacterial species isolated in a quantitative count of =/> 10(2) cfu/mL[2]. In typical practice, however, only one voided urine specimen is usually obtained and diagnosis is made with =/> 10(5) cfu/mL without obtaining a confirmatory repeat urine culture. Asymptomatic bacteriuria > 10(5) cfu/ml is treated with an antibiotic tailored to the susceptibility of the isolated organism [4]. The threshold for diagnosis and treatment of asymptomatic bacteriuria due to group B Streptococcus, during pregnancy, is lower at =/> 10(4) cfu/mL [11].

In this patient's particular case, a repeat culture revealed mixed flora. While the general interpretation of mixed flora is one that does not need to be followed up, it should be emphasized that the more appropriate interpretation is that the culture is contaminated and must be repeated especially in the case with a prior positive test. Further, positive cultures that have been treated should be repeated to ensure appropriate clearance of the pathogen. The timing for this is as yet undetermined [2]; however 1 to 2 weeks following completion of antibiotic therapy is reasonable. In our case, empiric treatment of multidrug resistant Klebsiella would have been inappropriate as it is normal skin flora; yet, ensuring that the culture was truly negative thereafter should have been a priority to reduce the subsequent morbidity experienced by the patient and staying true to the core value of antibiotic stewardship.

Proper management of ASB during pregnancy is critical to decrease the risks of maternal and neonatal adverse events. Suspicion for renal or perinephric abscess should arise when there are prolonged fever and flank pain, despite antimicrobial therapy [12]. When renal abscesses are less than 5 cm in diameter, antimicrobial therapy alone may be adequate initial management [13, 14]. When clinical symptoms persist after several days of antimicrobial therapy, percutaneous drainage of abscesses less than 5 cm should be considered. Patients with renal abscesses greater than 5 cm should be managed with percutaneous drainage in conjunction with antimicrobial therapy [14, 15]. In the antepartum period, ultrasound imaging should be considered to evaluate for structural abnormalities. Computed tomography with contrast enhancement is the ideal imaging for assessing a perinephric abscess and the extension of suppuration; however, in the antepartum period, the risks of fetal harm should be weighed [14, 16, 17]. Early surgical consultation is recommended for abscesses not amenable to drainage, anatomic abnormalities, or failed medical treatment.

For low-risk women with a negative urine test in their initial prenatal visit, rescreening for ASB is not indicated. For women with ASB in their prenatal screen or other risk factors, consideration should be given to urine cultures performed every trimester until the completion of pregnancy.

## Data Availability

Data sharing is not applicable to this article as no datasets were generated or analyzed for this case report.
